# Pneumatic computers for embedded control of microfluidics

**DOI:** 10.1126/sciadv.adg0201

**Published:** 2023-06-02

**Authors:** Siavash Ahrar, Manasi Raje, Irene C. Lee, Elliot E. Hui

**Affiliations:** ^1^ Department of Biomedical Engineering, University of California, Irvine, CA, USA.; ^2^ Department of Biomedical Engineering, California State University, Long Beach, CA, USA.

## Abstract

Alternative computing approaches that interface readily with physical systems are well suited for embedded control of those systems. We demonstrate finite state machines implemented as pneumatic circuits of microfluidic valves and use these controllers to direct microfluidic liquid handling procedures on the same chip. These monolithic integrated systems require only power to be supplied externally, in the form of a vacuum source. User input can be provided directly to the chip by covering pneumatic ports with a finger. State machines with up to four bits of state memory are demonstrated, and next-state combinational logic can be fully reprogrammed by changing the hole-punch pattern on a membrane in the chip. These pneumatic computers demonstrate a framework for the embedded control of physical systems and open a path to stand-alone lab-on-a-chip devices capable of highly complex functionality.

## Teaser

Programmable pneumatic computers built with fluidic circuits automate mixing and dilution procedures on integrated chips.

## INTRODUCTION

Embedded digital controllers are widely used for directing physical operations in integrated systems. Transduction between the electronic and physical realms can sometimes be unwieldy, however, requiring substantial mechanical hardware that can potentially dominate a system. It would thus be attractive to perform computing in alternative media that are more similar to the physical systems requiring control. This goal is being actively pursued in a variety of fields ranging from robotics (*1*–*3*) to biology (*4*–*7*), but the demonstrated control systems remain rudimentary compared to their electronic counterparts.

Microfluidics has produced powerful biochemical tools (*8*–*10*) and also established strong potential for digital computing (*11*–*17*), making it a promising platform for the development of nonelectronic embedded control. Prior work in this area has focused on reducing the number of interconnects required between the microfluidic chip and external electromechanical control hardware (*17*–*20*). However, complete elimination of off-chip control in favor of integrated control logic has not previously been possible. This would be a tremendous step toward the widespread use of lab-on-a-chip devices in point-of-care and limited-resource settings, which remains a long-standing but unrealized goal. For example, microfluidic devices with integrated control logic could potentially be cost-competitive with lateral flow assays, both being composed of a few injection-molded layers, while leveraging more complex liquid handling to gain advantages in speed, sensitivity, or functionality.

The finite state machine (FSM) is a widely used abstraction in digital control systems and can be implemented efficiently with a limited number of logic gates. It is a versatile computational model used in the control of a wide variety of systems including household appliances (*21*), video games (*22*), internet protocols (*23*), industrial processes (*24*), robots (*25*), and autonomous vehicles (*26*). In this work, we build FSM controllers out of microfluidic circuits and use these controllers to direct liquid handling processes on the same microfluidic chip.

## RESULTS

**Fig. 1.**
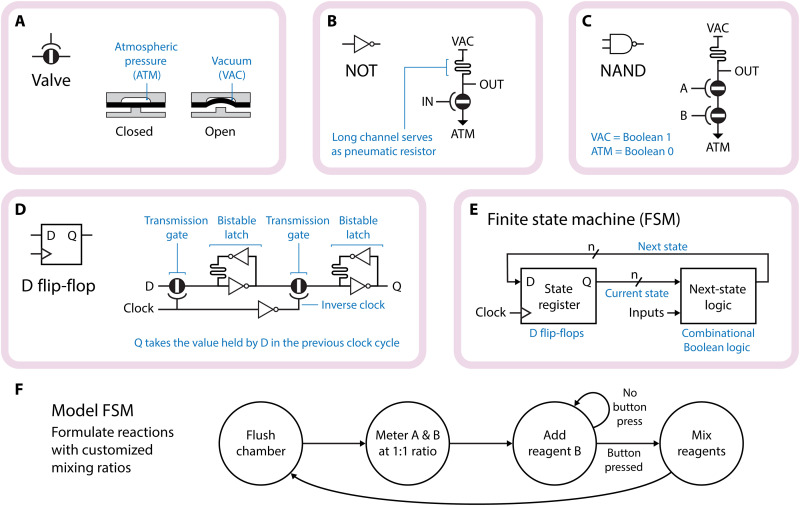
Building blocks of a pneumatic FSM. (**A**) Normally closed pneumatic membrane valve shown in cross section. The valves are three-terminal devices in which the channel is opened by applying vacuum to the gate. (**B** to **D**) Circuit diagrams for the fundamental Boolean logic gates and clocked memory element used in this work. (**E**) FSM architecture. Over the course of one clock cycle, the next state becomes the current state. The next state is then calculated before the beginning of another clock cycle. (**F**) Narrative state diagram for a model FSM similar to that implemented in Fig. 3. The arrows represent the rules governing the transitions from state to state. Once the states are assigned binary codes, the transition rules can be encoded into Boolean expressions and implemented as logic circuits in the next-state logic block.

Boolean logic was implemented by using circuits of pressure-actuated microfluidic membrane valves (*13*–*17*). Specifically, we used gates analogous to N-type metal-oxide-semiconductor logic, with normally closed membrane valves (*27*) forming the pull-down network and long microfluidic channels providing pull-up resistance (Fig. 1, A to C). Vacuum pressure represents Boolean 1, and atmospheric pressure represents Boolean 0. We have previously shown that this implementation achieves excellent pressure gain (*16*, *28*), which is critical for achieving robustness to noise and error-free data propagation through cascaded gates with fan-out.

An FSM consists of a set of program states, each with associated functional outputs, and a set of rules governing the transitions between different states. Figure 1F diagrams an FSM that allows the user to prepare reactions at customized mixing ratios. In a digital implementation, the system state is stored as a binary code in the state register, state transitions are calculated by combinational Boolean logic, and a system clock controls the timing of the transitions. We implemented state registers by using negative edge–triggered D Flip-Flops (*15*, *17*) in a clocked leader-follower configuration (Fig. 1D) to render the system robust against race conditions. The output of the state register (representing the current system state) is input into a combinational logic block along with any system inputs to calculate the next system state (Fig. 1E). During the subsequent clock cycle, the next state becomes the current state.

**Fig. 2.**
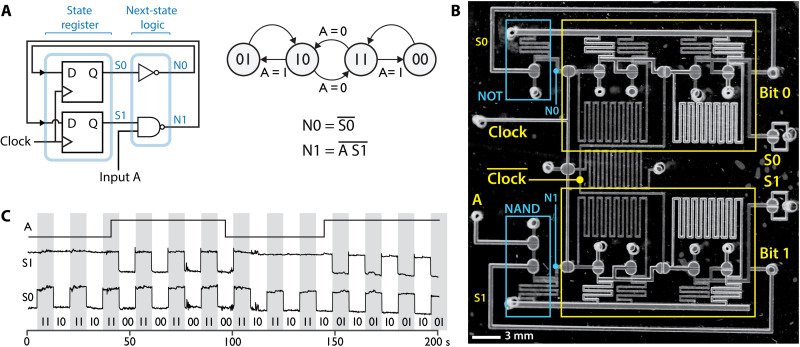
Pneumatic FSM. (**A**) Two-bit FSM block diagram and associated state transition diagram. Conditional branching is demonstrated by using an input, represented by A. The current state is represented by S0 and S1, while the next state is represented by N0 and N1. (**B**) Elements from the FSM block diagram of (A) are annotated in this image of the chip. Bit 0 marks the D flip-flop that stores S0. (**C**) Circuit timing diagram demonstrates that the device function matches the state transition diagram of (A). Waveforms are high for a binary value of 1 and low for a value of 0. While input A = 0, the system state oscillates between 11 and 10 (the middle loop of the state diagram). When input A = 1, the system oscillates between 11 and 00 (right loop) or between 10 and 01 (left loop), depending on the system state when A is switched. System state was measured via valves S0 and S1, but an indicator for A was not included on this device. Instead, switching of A was verbally narrated during video recording of S0 and S1, and the waveform for A was drawn to reflect the narrated state.

We began with a demonstration of conditional branching in a simple FSM with two bits of state memory and one input. This FSM program (state transition diagram shown in Fig. 2A) has two loops, one with the two bits oscillating in phase (00 or 11) and the other with the two bits oscillating in antiphase (01 or 10). The FSM remains in the current loop when the input is 1 and toggles to the other loop when the input is 0. The next state logic for this program can be implemented with one NOT gate and one NAND gate. The entire FSM was implemented with only 16 valves (Fig. 2B). An additional two valves were used as visual indicators of the current state. In this example, the input signal and clock were controlled electronically with off-chip solenoid valves and routed to the chip through pneumatic tubing. Circuit operation was filmed by adjusting the lighting to reflect differently from open and closed valves and then processed for presentation as a circuit timing diagram (Fig. 2C).

**Fig. 3.**
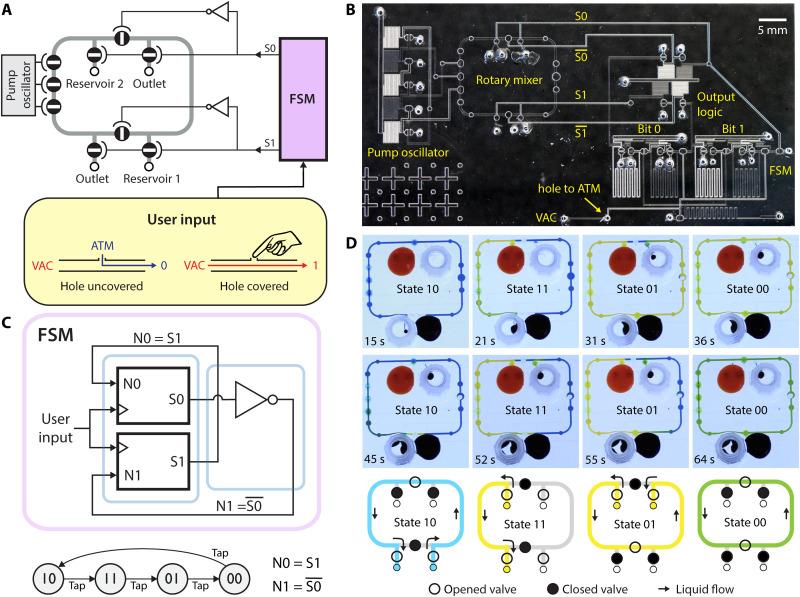
Embedded control of microfluidic liquid handling. (**A**) System-level diagram of rotary mixer with 2-bit FSM controller. The oscillator drives a peristaltic pump that continuously pushes liquid counterclockwise around the loop. The remaining valves are controlled by S0 and S1 to route flow as illustrated in (D). Push-button input is created by shorting a vacuum input to atmospheric ground through a hole that can be covered by the user. (**B**) Annotated image of the microfluidic chip. (**C**) FSM block diagram and state transition diagram. The user advances to the next state by tapping on the input button. The duration of state 01 is thus controlled by the user and determines the composition of the mix in state 00. (**D**) Images of the rotary mixer progressing through the different states. The flow path for each state is illustrated. In state 10, S1 = 1 and S0 = 0, thus opening three valves and closing three valves [compare with diagram in (A)] to route flow from reservoir 1 around the entire ring.

Next, we demonstrated embedded control by integrating an FSM with a rotary mixer (*19*, *29*). Here, the FSM controls valve settings in the mixer to direct a sequence of liquid handling operations (Fig. 3). Liquid flow is driven by a peristaltic pump controlled by an on-chip ring oscillator (*16*). The FSM was programmed to loop sequentially through four states (Fig. 3D). In the first state (10), the entire ring is filled from reservoir 1. In the second state (11), the left half of the ring is filled from reservoir 2. In the third state (01), the entire ring is filled from reservoir 2. In the fourth state (00), both reservoirs are sealed off and the contents of the ring are circulated for mixing. The mixing loop can be loaded with the contents of the two reservoirs at a variety of ratios, depending on whether the third state (01) is allowed to run to completion or is terminated early. This functionality is illustrated in Fig. 3D, where, in the first instance, the loop is mostly yellow during the mixing step, and, in the second instance, the loop is green.

Because the composition of the loop during the mixing step (state 00) is determined by the timing of the state transitions, we engineered an input method for the user to trigger these transitions (*17*). The system clock was directly connected to the vacuum supply, but the connection was shorted to ground by a port that is open to the atmosphere (Fig. 3A). While the port is uncovered, the clock signal holds steady at 0, but when the port is covered, the clock signal switches to 1. Thus, the user can advance the FSM to the next state simply by covering and then releasing the port with a finger. Please refer to movie S1. Notably, aside from the need for a vacuum supply, the entire system is self-contained on a single microfluidic chip (Fig. 3B). Liquids are pipetted directly onto the chip, and all timing signals are generated by the on-chip pneumatic oscillator or by user input via the on-chip button. This monolithic integrated system was built by using 31 valves: 17 for the FSM controller and 14 for the rotary mixer.

**Fig. 4.**
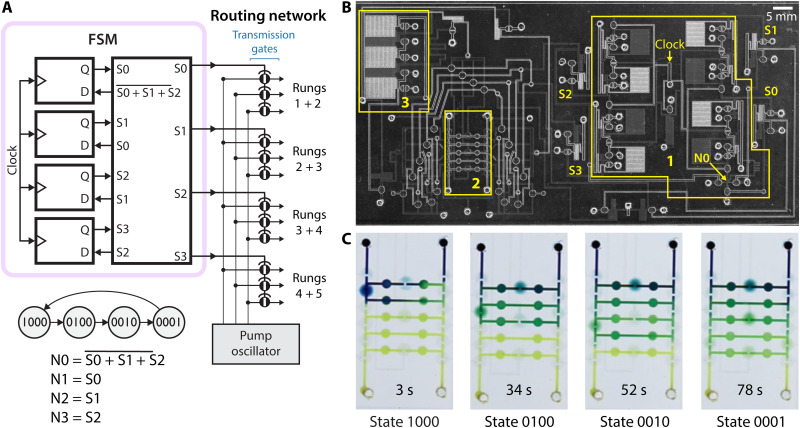
Autonomous control of serial dilution. (**A**) System-level diagram of the autonomous dilution ladder. A 4-bit FSM controls routing of peristaltic waveforms from the pump oscillator to the appropriate rungs of the dilution ladder. (**B**) Annotated image of microfluidic chip: FSM (box 1), dilution ladder (box 2), and ring oscillator (box 3). The pump control routing network makes up the remainder of the chip. (**C**) Time-lapse images of the 1:1 serial dilution process. Peristaltic pumping around each loop is driven by three valves actuated in a ripple pattern: one valve in the middle of a rung and another two on the far left and right. During each dilution step, additional unactuated valves along the far left and right seal off the rest of the rungs to create a closed loop between the two active rungs. The two smaller circles along each rung are not actuated but instead provide flexible windows to allow rung volume to expand and contract in response to peristalsis.

As a more complex example of embedded control, we chose to implement serial dilution, a ubiquitous multistep liquid handling procedure for creating a logarithmic range of solution concentrations. Serial dilution is a well-established method to derive quantitative data from simple assays with a binary readout (*30*, *31*), and making automated serial dilution available to consumers could potentially increase the number of medical conditions that can be diagnosed at home. We built upon our previously reported dilution ladder design (*32*), which accurately performs a sequence of 1:1 dilutions and preserves the entire dilution series. While the earlier work required off-chip solenoid valves under computer control, here, we used an on-chip FSM for embedded control (Fig. 4A). The dilution circuit consists of a set of liquid compartments arranged as rungs on a ladder. For each dilution step, two neighboring rungs are connected in a loop, and liquid is circulated until mixing is complete (Fig. 4C and movie S2). Timing signals for peristaltic pumping were generated by an on-chip ring oscillator and routed by the FSM to the appropriate rungs of the ladder for each stage of dilution. A 4-bit FSM was used to drive the four control lines of the ladder directly via one-hot encoding (each state has exactly one high bit), thus reducing chip real estate by avoiding the need for a demultiplexer. The autonomous serial dilution system (Fig. 4B) is the largest circuit presented in this work, using 81 valves: 38 for the FSM, 15 for the dilution ladder, 5 for the ring oscillator, and 23 valves for buffering and routing signals from the oscillator to the dilution ladder.

**Fig. 5.**
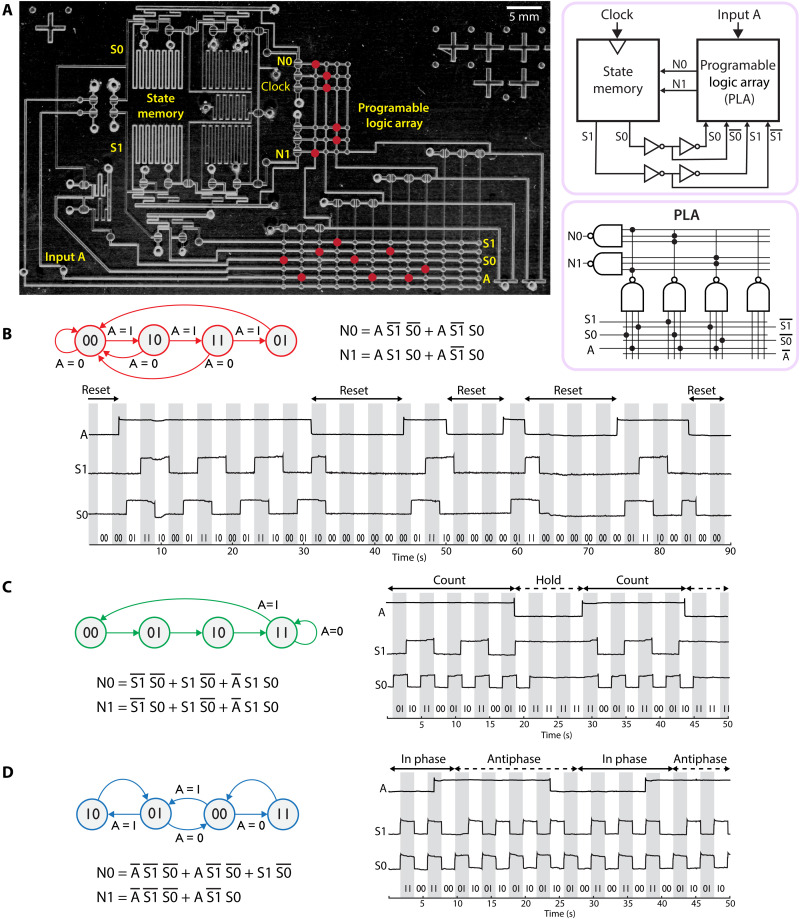
Programmable FSM encoded three ways. (**A**) Block diagram and annotated image of microfluidic chip. Red circles represent location of membrane bore holes to implement FSM of (B). (**B**) FSM steps through each state sequentially and resets to state 00 whenever input A = 0. (**C**) FSM steps through each state sequentially and pauses at state 11 if A = 0. (**D**) FSM states oscillate in phase when A = 0 and in antiphase when A = 1.

FSMs are defined by the rules that govern the transitions between states. Programmability thus entails the ability to specify these rules, which we achieved by using a programmable logic array (PLA) to implement next-state logic. The PLA is a user-configurable combinational logic circuit designed to allow different Boolean sum-of-product functions to be encoded. In the FSM design process, a state diagram is converted into a state transition table, which is then converted into a set of sum-of-product Boolean logic expressions (Fig. 5, B to D). These excitation equations were encoded into the microfluidic PLA by boring a set of holes in the membrane layer of the chip to define interconnects between the two circuit layers of the device (Fig. 5A). The PLA uses six inputs (2 state bits and 1 input bit, plus their inverses) and two outputs (2 next-state bits). Although a typical PLA is organized as a set of AND gates feeding into a set of OR gates, we chose the DeMorgan equivalent of NAND gates feeding into NAND gates, because NAND gates are more straightforward to implement in our technology. The PLA design was initially verified as a stand-alone device (figs. S1 and S2) before being integrated with a state register (Fig. 5A) to build a complete FSM. The system requires 35 valves: 18 for the PLA, 13 for state memory, and 4 valves for signal buffering. An additional four valves were used as signal indicators. We implemented three different FSM programs sequentially on a single chip. Reprogramming was accomplished by exchanging the membrane layer to obtain different interconnect hole patterns in the PLA circuit. The system was operated successfully at clock rates up to 5 Hz (movies S3 and S4). Circuit operation data for each FSM program are presented as timing diagrams in Fig. 5 (B to D).

## DISCUSSION

Lab-on-a-chip systems generally require substantial off-chip mechanical and electronic components to operate. While simple event staging can be achieved in stand-alone chips based on capillary flow (*33*, *34*), programmable operation of integrated valve networks requires control systems that greatly exceed both the cost and size of their associated microfluidic devices (*35*). On-chip microfluidic logic circuitry has already been deployed in commercial products to realize complex microfluidic functionality that otherwise would require an unmanageable number of fluidic connections between the chip and controller (*20*). Here, we take the next step, demonstrating a fully embedded control strategy that allows off-chip control to be dispensed with entirely. This approach produces monolithic systems-on-a-chip amenable to low-cost batch fabrication and exhibits compact form factors, greatly reducing the barriers to broad distribution of lab-on-a-chip systems. Pneumatic FSMs are also well suited to bring powerful capabilities to the embedded control of soft robotics, where pneumatic circuitry is already attracting substantial interest (*1*–*3*, *36*–*38*). Embedded systems of increasingly higher complexity will become possible as the circuit density of pneumatic logic continues to improve (*15*, *28*).

Integrated circuits that can be configured by customers after manufacturing are very important commercially, particularly in embedded systems. The microfluidic systems presented in this work consist of two glass layers containing etched channels, sandwiched around a flat membrane of silicone elastomer (*19*, *27*). For bulk manufacturing, the glass layers could be replaced by injection-molded plastic, and membrane through-holes could be defined by laser cutting (*28*). A single injection-molded design could thus be assembled with a number of different laser-cut membranes to implement a variety of different assays, each driven by a different control program. Programmability thus provides a strategy to produce a large assortment of niche products at a cost per device comparable to a mass-market product, through amortizing the cost of mold production across a set of dissimilar products. While vacuum supply is not widely available outside of laboratories and hospital rooms, the systems presented in this work can be powered by the vacuum from a small electrical pump or even a manual pump or syringe (*16*). Thus, this technology can support portable, stand-alone, biochemical processing systems.

A variety of nonelectronic computing approaches have been demonstrated at the proof-of-concept level, with the FLODAC fluidic microprocessor representing one of the most advanced efforts (*39*). However, information processing via physical systems faces inherent speed and density disadvantages compared to electronics, and systems like FLODAC simply could not compete with microelectronics. On the other hand, physical computing has distinct advantages for controlling physical systems, as transducers become unnecessary when control signals are calculated in the same physical modality as the system requiring control. It was in the control of air conditioning units, lawn sprinklers, and showerheads that fluidics was able to find some commercial success (*40*). Today, physical computing via pneumatic computers brings a programmable digital architecture miniaturized for the control of contemporary applications such as microfluidics and soft robotics.

## MATERIALS AND METHODS

### Fabrication

Devices were fabricated as previously described (*16*, *19*). Briefly, microchannels were defined in glass by photolithography and wet etching (50 μm deep). Ports were drilled through the glass to provide channel access. The channels were covered by a silicone sheet (254 μm), and vias were manually punched through the silicone under microscope observation. A second layer of glass channels was then laid on top of the silicone, again using a microscope to ensure proper alignment, and the layers were pressed together manually.

### Device operation

Switching of pneumatic inputs was accomplished via computer-controlled solenoid valves. Clock signals were often generated off chip to provide greater flexibility and control during circuit characterization. On-chip timing control by using oscillator circuits or manual push buttons was also used.

### Circuit analysis

Device operation was filmed with a digital camera (Canon EOS Rebel T1, 200-mm lens). Chips were positioned to maximize the difference in light reflection between open and closed valves. Circuit timing diagrams were extracted in MATLAB by plotting the normalized pixel intensity of selected regions of interest.
