# Supplementary material for: Pneumatic computers for embedded control of microfluidics
Source: Sci Adv. 2023 Jun 2;9(22):eadg0201. doi: 10.1126/sciadv.adg0201 (PMC10413662; doi:10.1126/sciadv.adg0201)
Supplement: Supplementary file 1 — Figs. S1 and S2 Legends for movies S1 to S4 [file sciadv.adg0201_sm.pdf]

Science Advances
AAAS

Supplementary Materials for

Pneumatic computers for embedded control of microfluidics

Siavash Ahrar *et al.*

Corresponding author: Elliot E. Hui, eehui@uci.edu

Sci. Adv. **9**, eadg0201 (2023)
DOI: 10.1126/sciadv.adg0201

The PDF file includes:

Figs. S1 and S2
Legends for movies S1 to S4

Other Supplementary Material for this manuscript includes the following:

Movies S1 to S4

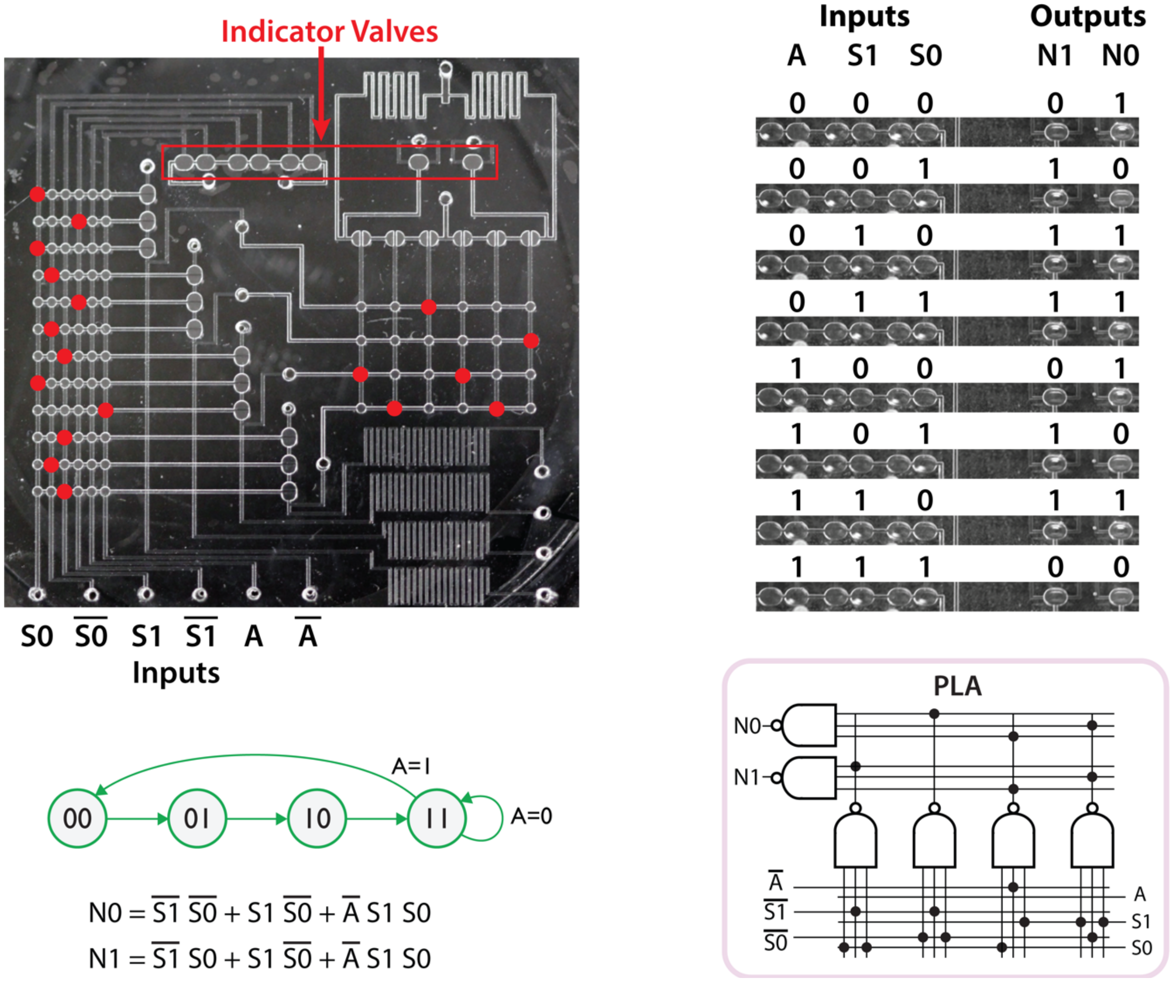

Fig. S1. Programmable logic array (PLA). Programmable combinational logic with 6 inputs and 2 outputs. The PLA is organized as four 3-input NAND gates followed by two 3-input NAND gates, which is equivalent to four AND gates followed by two OR gates. Bore holes through the membrane layer (indicated by the red dots) allow connections between horizontal channels on the first circuit layer and vertical channels on the second circuit layer. The bore hole positions were chosen to encode the sum-of-products excitation equations shown. Indicator valves allow the state of each input and output to be monitored simultaneously. Outputs were measured for all 8 possible input combinations, generating a truth table that verifies faithful implementation of the Boolean functions. The state diagram summarizes the system produced when this set of functions serve as the next-state logic in a finite state machine. Inputs represent the current state (S0, S1) and one input (A), along with the inverses of these three variables. Outputs represent the next state (N0, N1).

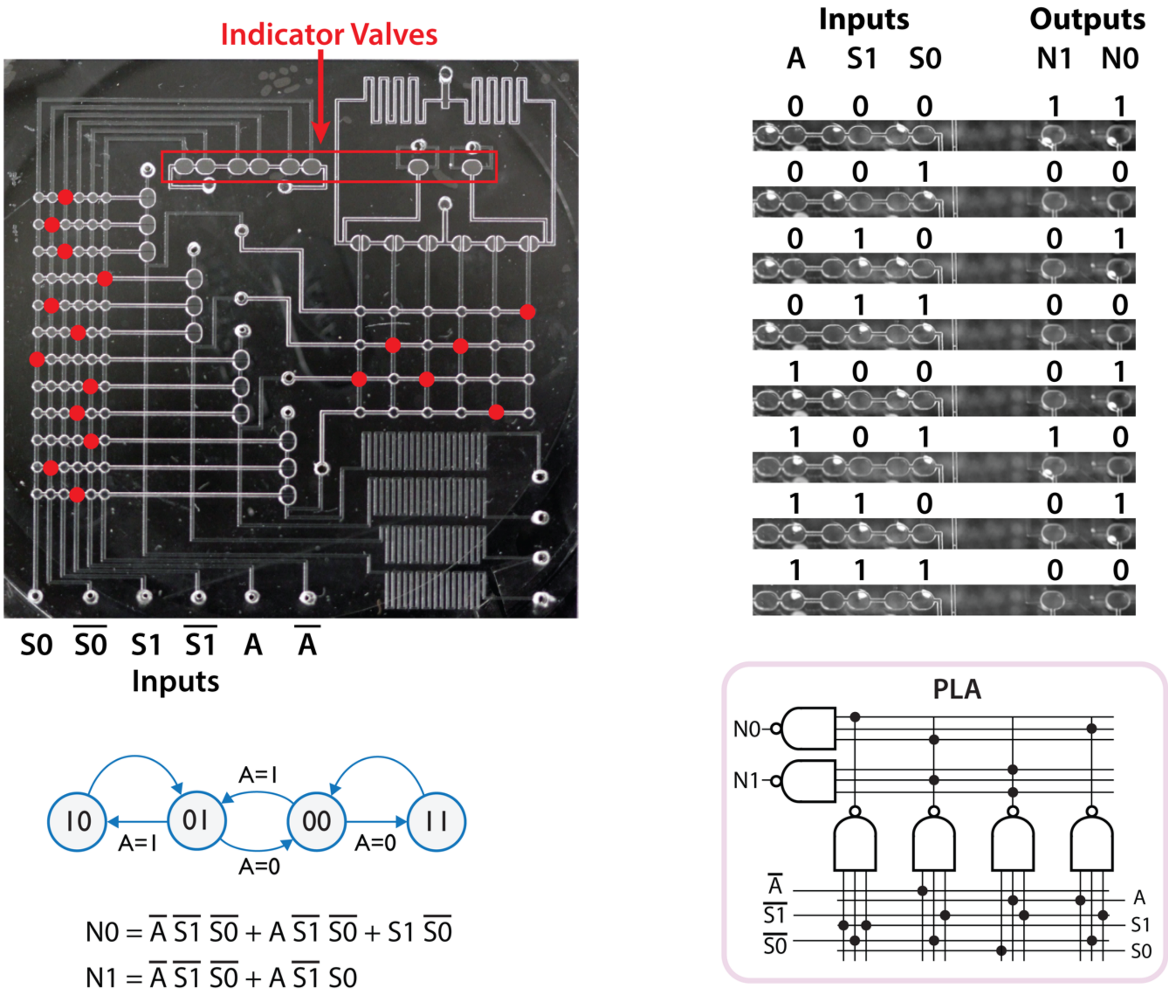

Fig. S2. Programmable logic array with alternative program. The same device as in Fig. S1, but with a different bore hole pattern (red dots) to encode a different set of Boolean functions. When employed in a finite state machine as the next-state logic, these Boolean functions implement the state transition diagram shown.

Supplementary Movie Captions

Movie S1. Generation of customized mixing ratios. Device contains embedded finite state machine controller with a button for user input. Tubing from off-chip provides only static vacuum, and the chip is otherwise fully self-contained. Peristaltic pumping is driven by a pneumatic oscillator circuit.

Movie S2. Autonomous control of serial dilution. Control of this complex liquid handling procedure is accomplished with an on-chip 4-bit finite state machine controller, which routes pump control signals from the on-chip oscillator to each successive rung of the dilution ladder. Vacuum power and the system clock signal are provided from off-chip. Movie is shown at 2x playback speed.

Movie S3. Programmable finite state machine encoded following Fig. 5B, running at 1 Hz clock.

Movie S4. Programmable finite state machine encoded following Fig. 5D, running at 5 Hz clock.
